# Role of Perinatal Stem Cell Secretome as Potential Therapy for Muscular Dystrophies

**DOI:** 10.3390/biomedicines13020458

**Published:** 2025-02-13

**Authors:** Serafina Pacilio, Sara Lombardi, Roberta Costa, Francesca Paris, Giovannamaria Petrocelli, Pasquale Marrazzo, Giovanna Cenacchi, Francesco Alviano

**Affiliations:** 1 Department of Biomedical and Neuromotor Sciences, Alma Mater Studiorum University of Bologna, 40126 Bologna, Italy; serafina.pacilio2@unibo.it (S.P.); sara.lombardi18@unibo.it (S.L.); r.costa@unibo.it (R.C.); francesca.paris6@unibo.it (F.P.); giovanna.cenacchi@unibo.it (G.C.); francesco.alviano@unibo.it (F.A.); 2 Department of Medical and Surgical Sciences, Alma Mater Studiorum University of Bologna, 40126 Bologna, Italy; giovannam.petrocell2@unibo.it; 3 Department of Biomolecular Sciences, University of Urbino Carlo Bo, 61029 Urbino, Italy

**Keywords:** muscular dystrophy, mesenchymal stem cells, perinatal secretome, cell-free therapy, extracellular vesicles, immunomodulation

## Abstract

Inflammation mechanisms play a critical role in muscle homeostasis, and in Muscular Dystrophies (MDs), the myofiber damage triggers chronic inflammation which significantly controls the disease progression. Immunomodulatory strategies able to target inflammatory pathways and mitigate the immune-mediated damage in MDs may provide new therapeutic options. Owing to its capacity of influencing the immune response and enhancing tissue repair, stem cells’ secretome has been proposed as an adjunct or standalone treatment for MDs. In this review study, we discuss the challenging points related to the inflammation condition characterizing MD pathology and provide a concise summary of the literature supporting the potential of perinatal stem cells in targeting and modulating the MD inflammation.

## 1. Muscular Dystrophies and Inflammation

Muscular Dystrophies (MDs) encompass a group of rare, inheritable genetic myopathies, characterized by progressive muscle weakness, muscle degeneration, and fibro-adipose tissue replacement [1]. According to the World Health Organization (WHO), MDs collectively affect approximately 1 in 3500 to 5000 live male births globally, with Duchenne Muscular Dystrophy (DMD) being the most prevalent form. These conditions impose significant economic and social burdens on patients, families, and healthcare systems. The pathogenetic mechanism of MDs is rarely due to a single gene defect; rather, it typically results from complex changes in multiple molecular networks [2]. One of the main challenges in managing MDs is the interplay between muscle degeneration and immune responses [3]. Inflammation plays a dual role in muscle homeostasis; indeed, even though it is essential for the activation and differentiation of muscle progenitor cells, during tissue repair, persistent inflammation can exacerbate muscle degeneration [4]. In MDs, the recurrent release of intracellular muscle components following myofiber damage triggers chronic inflammation, contributing significantly to disease progression [5]. MDs exhibit distinct immune profiles, driven by varying immunomodulatory molecules that regulate the immune reaction to muscle damage. Immune responses are typically characterized by tissue infiltration of macrophages, neutrophils, and both helper and cytotoxic T-lymphocytes [6]. For example, M1 macrophages, which are associated with a pro-inflammatory profile, promote the degradation of damaged muscle through the release of cytokines like Tumor Necrosis Factor (TNF-α), Interleukin 1β (IL-1β), and Interleukin 6 (IL-6) [7], facilitating the clearance of necrotic cells [8]. On the contrary, M2 macrophages release anti-inflammatory cytokines (e.g., IL-4 and IL-10) to suppress the inflammatory response and promote tissue repair [9,10]). This delicate balance between immune-mediated muscle degeneration and regeneration underscores the complexity of MD pathogenesis [3]. In Duchenne Muscular Dystrophy (DMD), muscle biopsies exhibit calcium deposits, varied sized and hypercontracted fibers, internal nuclei, fibrosis, and fatty infiltration [1]. The mutations in the dystrophin gene lead to chronic activation of the immune system [11] and both innate and adaptive immune responses play pivotal roles in exacerbating muscle damage [12]. Macrophages and cytotoxic T-cells release pro-inflammatory cytokines, such as TNF-α and Interferon γ (IFNγ), perpetuating muscle degeneration and fibrosis [13]. Therapeutic interventions targeting macrophages, TGF-β signaling, and fibrosis have shown promise in preclinical models of DMD [12]. Moreover, the mitochondrial component in DMD pathogenesis is increasingly recognized as a critical factor in disease progression. Dysfunctional mitochondria contribute to Oxidative Stress, impaired calcium homeostasis, and energy deficits, exacerbating myofiber damage. This mitochondrial dysfunction is intricately linked to chronic inflammation and the activation of immune responses, further worsening muscle degeneration [14]. Additionally, Dubinin et al. [15] identified the mitochondrial permeability transition (MPT) pore as a key contributor to mitochondrial dysfunction in dystrophin-deficient mdx mice and demonstrated the therapeutic potential of alisporivir, a non-immunosuppressive MPT pore inhibitor, in preserving mitochondrial function and alleviating cardiomyopathy in DMD. Limb-girdle muscular dystrophies (LGMDs) also involve immune activation. In LGMD, R5 and R9 inflammation is a prominent feature, and patients often respond to corticosteroid treatment, suggesting that immune cells such as B-cells, CD4^+^, and CD8^+^ T-cells contribute to disease pathology [16,17]. Dysferlinopathies, that feature inflammatory infiltrates, myonecrosis, and regeneration, including Miyoshi myopathy and LGMD type 2R are marked by defective membrane repair mechanisms, leading to immune cell infiltration, particularly macrophages [18]. Inflammatory cytokines such as TNF-α and iNOS are elevated, and NF-κB signaling is implicated in disease progression [19]. Notably, the complement system plays a distinct role in dysferlinopathies, with the Membrane Attack Complex (MAC) localizing to muscle fibers, an absent feature in DMD, indicating disease-specific immune mechanisms [20]. Congenital Muscular Dystrophies (CMDs) exhibit inflammation like in DMD, with macrophages dominating the inflammatory infiltrates [21] and marked dystrophic changes with striking fatty infiltration. Lamininopathies, such as Merosin-Deficient Congenital Muscular Dystrophy (MDC1A), are associated with early immune cell invasion and activation of TLR and NF-κB signaling pathways [22]. Laminin α-2-deficient dystrophy shows fatty infiltration, with complete absence or patchy reduction in laminin α-2. In Facioscapulohumeral Muscular Dystrophy (FSHD), around 30–40% of patients exhibit muscle inflammation, with macrophages and T-cells accumulating in the muscle tissue [23]. Other MDs, such as Emery-Dreifuss Muscular Dystrophy (EDMD) and Oculopharyngeal Muscular Dystrophy (OPMD), also involve immune responses, though to varying degrees [24]. In EDMD, which displays fiber size variation, internal nuclei, fibrosis, and altered myofibril orientation [25], macrophages and T-cells have been observed in muscle tissues, and inflammatory markers like TNF-α and IL-6 in muscle biopsies are elevated [26]. In OPMD, the peculiar accumulation of nuclear aggregates in muscle fibers activates an immune response, with macrophages and CD8^+^ T-cells contributing to muscle inflammation and fibrosis [25,27]. Myotonic Dystrophy (MD) type 1 and 2, although primarily associated with muscle wasting and multi-systemic involvement, also show evidence of immune activation [28]. Muscle biopsies demonstrate irregularity in muscle fiber size, rows of internal nuclei, muscle fibrosis, and myofibril orientation perpendicular to the muscle fiber. However, elevated muscle-related levels of pro-inflammatory cytokines and macrophage infiltration suggest a role for the immune system in developing muscle degeneration and fibrosis, especially in cardiac and respiratory muscles [29]. A description of the main characteristics, molecular mechanisms, and inflammatory involvement of the mentioned MD is provided in Table 1.

Across these diverse forms of MD, the chronic activation of immune responses and the resulting inflammation and fibrosis are common pathological features [30]. While treatments targeting inflammatory pathways are primarily symptomatic, they may become curative if capable of modulating the molecular alterations underlying the disease [31]. Cell-based therapies, anti-inflammatory drugs, and immunomodulatory agents are at the forefront of ongoing research aimed at mitigating the immune-mediated damage in these diseases [32]. Pharmacological interventions remain a cornerstone in the management of MDs, particularly Duchenne Muscular Dystrophy (DMD). Glucocorticoids, such as prednisone, prednisolone, deflazacort, and vamorolone, are commonly employed for their anti-inflammatory properties. These agents function by inhibiting the NF-κB pathway, leading to prolonged ambulation, improved pulmonary function, and delayed cardiomyopathy onset in DMD patients [33]. Despite their benefits, long-term glucocorticoid use is associated with adverse effects, including growth retardation, bone demineralization, and metabolic disturbances. Consequently, there is a pressing need for alternative therapies with improved safety profiles. Emerging pharmacological strategies focus on novel agents such as tamoxifen, which has demonstrated potential in modulating muscle degeneration pathways [34]. Additionally, gene therapy approaches, including exon skipping and stop codon readthrough, aim to restore dystrophin function, offering promising avenues for disease modification [35]. While these innovative therapies are under investigation, their long-term efficacy and safety remain to be fully established. In particular, by targeting and repairing specific genomic sequences, CRISPR-Cas9 holds the potential for long-term disease amelioration [36]. Despite its promise, it faces significant challenges, including off-target effects, delivery inefficiencies, and ethical concerns surrounding germline editing. Additionally, this approach does not directly address the inflammatory milieu that perpetuates muscle damage, highlighting the need for complementary therapies [37]. Moreover, the role of gut microbiota seems to be relevant as a potential means of therapeutic intervention. Gut microbiota are increasingly recognized for their influence on systemic inflammation and muscle homeostasis [38]. Dysbiosis, or an imbalance in the microbial community, has been implicated in exacerbating inflammation in MDs. Interventions aimed at restoring a healthy microbiota through probiotics, prebiotics, or dietary modifications have shown potential in reducing inflammatory markers. Moreover, the complex interplay between gut and muscles requires further elucidation to optimize therapeutic outcomes [39]. Finally, recent findings highlight the therapeutic potential of the stem cell secretome in modulating inflammation and promoting tissue repair in various congenital pathologies. Tung et al. [40] describe the diverse bioactive components of the secretome, including cytokines, growth factors, and extracellular vesicles, which collectively contribute to its regenerative and immunomodulatory properties. These mediators can target pathological mechanisms, offering a foundation for exploring similar strategies in MD. Given the growing potential of perinatal stem cells in inflammation modulation, this review aims to highlight the challenges associated with the inflammatory conditions characterizing MDs pathology and explore how perinatal stem cells and their secretome could contribute to their regulation.

## 2. Skeletal Muscle Recovery: Challenges and Therapeutic Strategies

Chronic inflammation remains a major driver of muscle damage [2,41]. Reactive oxygen species (ROS) are key contributors to the process, making them ideal targets for therapeutic intervention [42], acting as both central players and byproducts of the inflammatory process. ROS production is triggered by mutations in muscle-regulating proteins, leading to myofiber damage and the accumulation of inflammatory cells. In Muscular Dystrophies, non-selective channels like connexin hemichannels increase cytosolic Ca^2+^, activating proteases and mitochondrial dysfunction. ROS overproduction leads to Ca^2+^ leak, oxidation of lipids, proteins, and DNA, and disruption of NF-κB and Nrf2 signaling, contributing to muscle degeneration [24]. Anti-inflammatory and antioxidant agents, particularly those with fewer side effects compared to glucocorticoids, are essential for long-term management [43]. Recent advances in stem cell-based therapies, including induced pluripotent stem cells (iPSCs), have generated optimism for treating MDs [32,44,45].A list of stem cells involved in cell therapy approaches for MD is shown in Table 2. However, clinical translation of stem cell-based therapies remains limited by issues such as immune rejection and the reduced viability of transplanted cells derived from the hostile inflammatory environment [46]. A diverse range of cell types has been investigated for their myogenic potential, including myoblasts [47], muscle precursor cells, CD133^+^ cells [48], Bone Marrow Mononuclear Cells (BM MNCs) [49], mesoangioblasts [50], and Mesenchymal Stem Cells (MSCs) ([51] Cells such as Side Population (SP) cells, Muscle-Derived Stem Cells (MDSCs), myogenic-endothelial progenitors, and pericytes have shown the capacity to differentiate into skeletal muscle tissue in both in vitro and in vivo studies [52,53]. However, due to the progressive replacement of muscle with connective and adipose tissue occurring in advanced MD stages, most therapies would be ineffective if started late, making early intervention essential. As efforts to optimize cell-based therapies, emerging research has shifted focus toward the paracrine effects of stem cells, particularly their secretome [54]. The secretome, comprising growth factors, cytokines, and extracellular vesicles (EVs) released by stem cells [55], has demonstrated significant pro-therapeutic potential. Rather than relying solely on cell replacement, leveraging the bioactive molecules within the secretome may provide an alternative approach to modulating the muscle environment, reducing inflammation, and promoting tissue regeneration [56]. The secretome’s capacity to influence the immune response and enhance tissue repair, without the risks associated with cellular transplantation, has been proposed as an adjunct or even a standalone therapeutic strategy in MD treatment. This review will focus on recent advances in the use of secretome derived from perinatal tissues, particularly the amniotic membrane and umbilical cord, for treating MDs acting on their inflammatory features.

## 3. Stem Cell Secretome: Immunomodulatory and Potential Therapeutic Features

A secretome consists of soluble paracrine agents secreted by cells in free form or in EVs, including cytokines, growth factors, and regulatory nucleic acids, which play a key role in tissue regeneration [57]. Research shows that the therapeutic benefits of stem cells are mediated by the factors they secrete, highlighting the secretome as a promising cell-free alternative to direct stem cell therapy [58]. It has shown potential in promoting tissue repair and modulating the immune system [59], with promising results in treating skeletal muscle injuries, spinal disc degeneration, skin wounds [60], liver disorders [61], cardiovascular diseases [47,62], neurodegenerative conditions [63], and osteoarthritis [64].

### 3.1. The Perinatal Cells Secretome

Perinatal tissues, derived from term placentas and fetal annexes, are exposed to high metabolic demands that increase mitochondrial activity and generate Reactive oxygen species (ROS), leading to Oxidative Stress (OS) and inflammation. Specifically, from the innermost layer on, these tissues include the amniotic fluid, amniotic membrane, chorionic membrane, chorionic villi, umbilical cord (including Wharton’s jelly), and placental basal plate (comprising both maternal and fetal cells [65]. Several in vitro studies have demonstrated that perinatal cells interact with both the innate and adaptive immune systems, targeting T and B lymphocytes, macrophages, dendritic cells, neutrophils, and natural killer cells [66]. Mesenchymal Stem Cells (MSCs) and Amniotic Epithelial Cells (AECs) derived from perinatal tissues exhibit a remarkable capacity to modulate the immune response, making them attractive candidates for therapeutic use [67]. AECs from the placenta, known for their immunoprivileged phenotype, play a critical role in maintaining maternal–fetal tolerance [68,69]. Their low expression of HLA class IA, absence of HLA class II, and expression of non-classical molecules, such as HLA-G, HLA-E, and HLA-F, enable them to suppress natural killer (NK) cell cytotoxicity and modulate dendritic and T cell activity [70]. These cells also secrete anti-inflammatory factors such as IL-10 and Prostaglandin E2 (PGE2), promoting an anti-inflammatory macrophage phenotype while counteracting fibrosis. Indeed, the immunosuppressive capability of the AEC-derived secretome seems to be a promising agent for reducing inflammation and tissue damage in MDs [71]. Similarly, MSCs are known to exhibit a therapeutic responsive polarization, allowing them to adopt either pro- or anti-inflammatory phenotypes depending on their microenvironment [72]. Preconditioning MSCs with pathogen-associated molecular patterns (PAMPs) like lipopolysaccharides (LPSs) enhances their anti-inflammatory effects, as demonstrated by the production of EVs with superior immunomodulatory properties [73]. Moreover, Wharton’s Jelly-MSCs (WJ-MSCs) share characteristics with bone marrow-derived MSCs (BM-MSCs) and Embryonic Stem Cells (ESCs) [72,74]. Compared to adult MSCs, WJ-MSCs exhibit superior “stemness” due to minimal exposure to environmental factors and genetic alterations. These qualities, along with their reduced teratoma risk, make them promising candidates for clinical use [75]. WJ-MSCs’ secretome plays critical roles in cellular homeostasis, anti-inflammation, and immunomodulation [76,77]. Recent studies [78] indicate that the secretome of WJ-MSCs enhances neutrophil function and longevity, with potential therapeutic applications in neutropenia or chronic granulomatous disease [78]. For instance, WJ-MSCs preconditioned with LPS produce EVs that accelerate wound healing and reduce Oxidative Stress in diabetic models [79]. This highlights the potential of secretome for decreasing inflammation and enhancing tissue regeneration without requiring direct cell transplantation. The culture of MSCs in 3D settings has emerged as a promising strategy to optimize their paracrine activity [80]). By replicating the in vivo niche and promoting intercellular interactions, 3D spheroid cultures significantly enhance the secretion of key immunomodulatory factors such as transforming growth factor-beta 1 (TGF-β1), IL-6, Tumor Necrosis Factor-Stimulated Gene-6 (TSG-6), and PGE2 [81]. Moreover, dynamic 3D cultures yield a higher number of EVs with enhanced anti-inflammatory effects, reducing CD8^+^ T cell proliferation and Oxidative Stress while promoting wound closure. The 3D spatial organization of MSCs not only maintains their low immunogenicity but also increases their therapeutic potential, making the 3D aggregate method an exciting avenue for future clinical applications [82]. In addition to their immunomodulatory properties, the antioxidant potential of the stem cell secretome is crucial in mitigating the effects of Oxidative Stress (OS), which is a significant contributor to muscle degeneration in dystrophic conditions. Perinatal cells, particularly AECs and MSCs, are susceptible to OS-induced damage which leads to cellular senescence, DNA damage, and impaired cell function [83]. Studies have demonstrated that the antioxidant components of secretome can counteract ROS, protect cell membranes, and prevent apoptosis [84,85]. Optimizing culture conditions and employing antioxidant strategies may further enhance the therapeutic efficacy of perinatal-derived secretomes [86]. Clinical trials exploring the use of the WJ-MSC secretome in various conditions, including type 1 diabetes mellitus (T1DM) [87], have shown promising results [88]. For instance, early-phase trials suggest that secretome-based therapies may help in restoring insulin production in newly diagnosed T1DM patients [89]. These findings underscore the potential of the stem cell-derived secretome as a versatile therapeutic tool, extending beyond traditional cell therapy to treat a range of degenerative diseases, including MDs. As the field of regenerative medicine evolves, the use of cell-free therapies such as the stem cell secretome offers a compelling alternative to conventional methods [90]. By harnessing their immunomodulatory and antioxidant properties, secretomes may provide a safer, ethically favorable, and effective treatment option for managing inflammation and Oxidative Stress, key drivers of muscle degeneration in dystrophic patients. This insight has spurred interest in engineering or preconditioning MSCs to enhance their therapeutic potential, particularly by refining their secretory profile, especially the extracellular vesicles (EVs) [91].

### 3.2. Extracellular Vesicles: Main Players of the Stem Cell Secretome

Extracellular vesicles (EVs) are bilayer membrane-bound structures rich in bioactive molecules like lipids, nucleic acids and proteins. EVs are generally classified into three types: exosomes (Exo), microvesicles (MVs), and apoptotic bodies, differentiated by their origin, size, cargo, function, and mechanisms of release [92]. Today, EVs are recognized as main mediators of intercellular communication, extending beyond traditional cell–cell interactions and secreted molecules. EVs influence recipient cells by either interacting via ligand–receptor mechanisms or by fusing and transferring their bioactive content directly into the target cell cytosol, modifying its physiological state. EVs can also release their contents into the extracellular environment, allowing further signaling modulation [93]. EVs can be isolated from multiple biological fluids like blood, urine, plasma, breast milk, and amniotic fluid and tissues such as brain and lung tumors, as well as perinatal sources like the placenta [94]. Under normal conditions, EVs contribute to the regulation of physiological processes like immune responses, coagulation, angiogenesis, apoptosis, and cellular homeostasis [95]. On the other hand, they also play a significant role in the onset and progression of various diseases, including cancer, neurodegeneration, infections, and cardiovascular disorders [96,97]. Owing to their immunomodulatory properties, EV-based therapies are being investigated for the treatment of inflammatory diseases, autoimmune conditions, and cancer [98]. A deeper understanding of the mechanisms through which EVs exert their immunomodulatory effects is currently being investigated to specifically identify their bioactive cargo and target cells [99].

The role of EVs as the sole mediators of the immunomodulatory effects of the secretome has been disputed by other studies pointing out that the immunomodulatory effect is exerted by the secretome in toto and not by secreted factors directly conveyed by EVs. Papait et al. [100] reported that the immunomodulatory effects of the hAMSCs secretome are primarily due to factors in the CM rather than the EVs themselves, which, although internalized by immune cells, did not show significant effects at their original concentrations. Others have shown that EVs have a reduced impact compared to their parental MSCs [101,102], or an immunosuppressive action on T cells [103], and some even found no effect of EVs at all [104]. Similarly, research investigating the impact of MSC-derived EVs on B cells has produced conflicting findings [105]. Indeed, Carreras-Planella et al. reported that MSC-EVs were unable to stimulate naïve B cell expansion or decrease memory B cells. While MSC-EVs induced CD24ʰⁱ CD38ʰⁱ B cells at levels comparable to MSCs, they did not generate true regulatory B cells (Bregs) as they failed to produce IL-10. These findings suggest that MSCs influence B cell modulation through soluble factors other than EVs. In Conforti’s study [101], MSC co-culture led to a statistically significant rise in IL-10 and TGF-β, alongside a reduction in GM-CSF and IFN-γ, compared to EVs incubation. Their findings suggest that EVs exhibit a diminished immunomodulatory impact on T-cell proliferation and antibody production in vitro, relative to their cellular counterpart, not provoking a significant clinical response in most GvHD patients. These discrepancies may arise from methodological differences that make comparisons across studies challenging.

## 4. Advances in Secretome from Perinatal Cells for MDs Treatment

Perinatal cells and their secretome have shown strong immunomodulatory potential [106,107], a property closely linked to their ability to support tissue regeneration [107], especially in conditions characterized by acute or chronic inflammation, such as MDs. A recent study by Sandonà [108] demonstrated that the human amniotic mesenchymal stem cell (hAMSC) secretome directly impacts the muscle stem cell (MuSC) niche by enhancing progenitor cell proliferation and differentiation, typically compromised in DMD muscles. By employing a neutral sphingomyelinase inhibitor to block EV release from hAMSCs, they revealed distinct roles for the hAMSC secretome and its EV fraction in muscle regeneration, indicating that freely secreted factors from the conditioned medium (CM) primarily support MuSC proliferation, as evidenced by a higher increase in nuclei count, while EVs are more effective in driving MuSC differentiation, as shown by a higher myotube fusion index. Significantly, local treatment with hAMSC-derived EVs greatly facilitated muscle regeneration in dystrophic mice, reducing muscle fibrosis and improving muscle function. This treatment enhanced myofiber size, and led to increased formation of new myofibers and to a greater number of proliferating MuSCs within the fibers themselves. Additionally, conditioned medium and exosomes from placental MSCs showed similar enhancement of myoblast differentiation and reduced fibrogenic gene expression in DMD models, though placental MSCs are less specifically characterized compared to amniotic MSCs [109]. This study also explores the factors mediating the hAMSC secretome’s regenerative effects. Interestingly, the CM of hAMSCs is rich in metalloproteinase (MMP) inhibitors (TIMP1 and TIMP2), which counteract MMPs involved in DMD progression. Moreover, CM hAMSC contains Growth Differentiation Factor 15 (GDF15), a myomitokine with a role in energy metabolism but with a still debated impact on muscle diseases. Moreover, hAMSC-derived EVs carry miRNAs that have a critical role in muscle differentiation and regeneration, such as miR-26a, which targets Smad1 and Smad4 in the TGF-β/BMP pathway, a well-known inhibitor of differentiation, as well as miR-214, which negatively regulates Ezh2 protein, accelerating skeletal muscle cells differentiation. These results align with other studies showing that EVs from fetal and placental stem cells can reduce fibrosis and support muscle repair in MuSCs [108]. Another study showed that WJ-MSC EVs improved angiogenesis and myogenesis, reduced fibrosis, and directed inflammation aimed to muscle repair, with a notable increase in M2-like pro-regenerative macrophage numbers compared to controls in a volumetric muscle loss murine model [110]. Bier et al. [109] showed that placenta-derived exosomes have potential for treating DMD. They studied the effects of placenta-derived mesenchymal stem cells (PL-MSCs) and their exosomes on muscle cells from both healthy controls and DMD models. Treatment with PL-MSCs or their exosomes improved muscle cell differentiation, reduced fibrosis-related genes, and increased utrophin levels, which may help to restore muscle function. The therapeutic effects were linked to exosomal miR-29c, which promotes muscle repair and reduces fibrosis. By silencing miR-29c, these benefits were diminished, highlighting the important role of this miRNA. These results confirmed the previous ones by Nakamura [111] demonstrating that MSC exosomes promote myogenesis and angiogenesis in vitro, and muscle regeneration in an in vivo model of muscle injury, an effect partly mediated by miRNAs, such as miR-494. In addition, another relevant example of exosomes-derived miRNA activity in the MD context has been shown by Sandonà et al. [54]. They demonstrated that EVs released by mesenchymal cells like fibro–adipogenic progenitors (FAPs) mediate miRNAs transfer to MuSCs. In addition, when dystrophic FAPs are treated with HDAC inhibitors (HDACis), they release EVs containing increased levels of specific miRs, such as miR-206, a muscle-specific miR, that cooperatively target key biological processes related to muscle regeneration, fibrosis reduction, and inflammation control. In DMD patients and “mdx mice”, exposure to HDACis raises miR-206 levels in FAP-released EVs, enhancing muscle regeneration and reducing fibrosis. When EVs are transplanted into dystrophic muscles, they activate and expand MuSCs, boosting regeneration while inhibiting both fibrosis and inflammation. Inhibiting individual miRs, such as miR-206, reveals that it is essential for EV-induced MuSC expansion and muscle regeneration. Furthermore, the combined activity of HDACi-induced miRs contributes to the overall beneficial effects of these EVs. In the context of inflammation, Noonin and Thongboonkerd [112] demonstrated that miR-181c in exosomes from human Umbilical Cord MSCs (hUCMSCs) reduces TLR4 expression and NFκB activation, lowering pro-inflammatory cytokine levels [113]. Fibrosis, a mechanism for which it is necessary to focus on muscular disorders, could find its remedy, as demonstrated by Hodge et al. [61]. They found out that amniotic epithelial cells (hAECs) and their CM reduced liver fibrosis and injury in a mouse model. Eventually, besides secretomes or ECVs alone, co-cultures of perinatal stem cells and myoblasts also showed a positive effect in treating myopathies-related pathological effects. Kwon et al. [114] highlighted the protective role of hWJ-MSCs in preventing muscle cell death. WJ-MSCs significantly reduced apoptosis in mouse skeletal muscle myoblasts (C2C12 cell line) under serum deprivation conditions, knowing that antibody analysis revealed high levels of chemokine XCL1 secretion. XCL1 treatment effectively inhibited apoptosis in both serum-starved and lovastatin-treated C2C12 cells, but not in other cell lines. Knockdown of XCL1 in WJ-MSCs confirmed its pivotal role in this anti-apoptotic effect. Additionally, XCL1 treatment improved muscle defects in a zebrafish myopathy model, suggesting its potential as a therapeutic agent for muscle diseases [114]. Furthermore, Kono et al. confirmed the paracrine effect of mouse MSCs on the inflammatory response of LPS-activated C2C12 cells. IL-6 production from LPS-activated C2C12 cells was significantly increased after co-culturing with MSCs. Moreover, IL-6 and Inducible Nitric Oxide Synthase (iNOS) mRNA expression was notably upregulated in C2C12 cells cocultured with MSCs, while TNF-α and IL-1β mRNA expression was reduced.

## 5. Discussion and Future Outlooks

**Figure 1:**
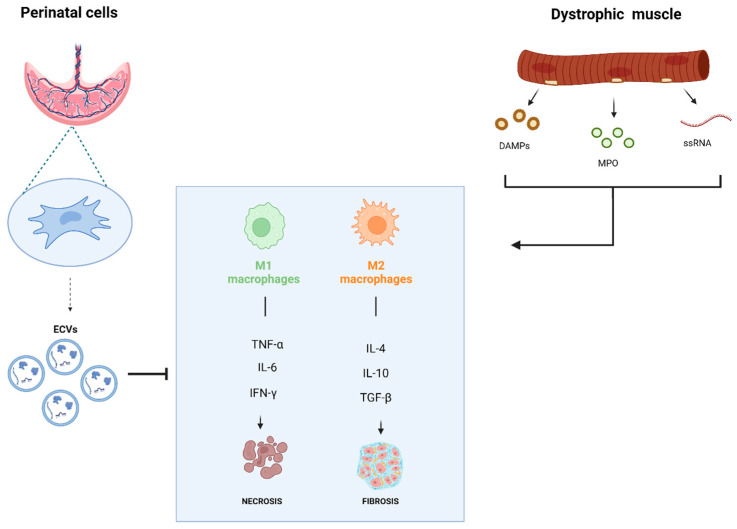
Interplay between perinatal cells and the dystrophic muscles. The effect of dystrophic muscle on macrophage M1/M2 activation and associated cytokines production and the influence of perinatal cell secretome.

Recent research revealed how the immune system plays a significant role in MDs, showing insights into the disease mechanisms behind MDs and the interplay between muscle and the immune system, opening new therapeutic avenues for possible treatments of MDs. Understanding immune system interactions with dystrophic muscle has led to the development of new treatment strategies beyond traditional immunosuppressants regimens. To date, MuSC-derived myoblasts, mesoangioblasts (MABs), CD133^+^ cells, MSCs, and CDCs have been evaluated in clinical trials for MDs. Despite some positive indications in terms of safety or functional/histological recovery, these therapeutic options appear still preliminary. As efforts to enhance cell-based therapies continue, emerging research has increasingly focused on the paracrine effects of stem cells, especially how their secretome can influence the immune system. This literary review aimed to analyze the immunomodulatory and antioxidant potential of perinatal stem cell-derived secretomes and their EVs as potential therapeutic approaches for MDs treatment. The stem cell secretome derived from perinatal tissues is already known to support tissue regeneration, relying on its adaptable immunomodulatory activity. A graphic summary of this review study is represented in Figure 1. The amniotic membrane and umbilical cord are valuable sources of MSCs and AECs, excellent candidates for obtaining immunomodulatory secretomes. For example, the secretome from hAMSCs enhances MuSC function, with free factors supporting MuSC proliferation and EVs promoting differentiation, muscle regeneration, reduced fibrosis, and increased myofiber formation in dystrophic mice [108]. In addition, EVs from hWJ-MSCs improved angiogenesis and myogenesis and reduced fibrosis in muscle injury models [110]. They also increased M2-like macrophages, which are associated with tissue repair. Exosomal miRNAs (e.g., miR-29, miR-26, miR-214) play a significant role in enabling these processes [108,109,111]. However, there are conflicting findings regarding the impact of purified EVs compared to their parental cell effects, with some research suggesting that EVs are less effective or even non-immunosuppressive on certain immune cells [101,102,105]. To advance clinical applications and next-generation stem cell-EV therapy, the ideal stem cell source should be chosen according to its paracrine potential and its ease of isolation. Perinatal stem cells like AECs and WJ-MSCs, which are isolated from delivery waste clinical material, along with the lack of ethical concern, and consistent expansion and cryopreservation, proved to be optimal candidates over adult MSCs and Embryonic Stem Cells as sources for future EVs-based paracrine therapy. Nowadays, the clinical applications of EVs remain challenging due to the lack of standardized protocols to produce vesicles for use in human therapy. There are open debates about the diversity and preparation of stem cell EVs and consequently about the selection of methods for EV isolation and purification. Future challenges include the availability of standardized quality tests, and the precision improvement of in vitro and in vivo functional assays will be essential. With ongoing advancements, these factors could greatly impact the consistency and reliability of cell-free therapies, soon offering renewed hope to patients affected by skeletal muscle disorders.

## Figures and Tables

**Table 1:** Muscular Dystrophies key features, mechanism, and related-immune responses.

Muscular Dystrophy	Key Features	Mechanisms	Immune Responses
Duchenne Muscular Dystrophy (DMD)	Muscle weakness, fibrosis, fat deposits	Dystrophin mutations lead to chronic inflammation	TNF-α, IFNγ release by cytotoxic T-cell and macrophages
Limb Girdle MDs (LGMDs)	Weakness in pelvic and shoulder muscles	Sarcolemmal proteins tirigger to inflammation	T-cells, B-cells, and macrophages involved
Dysferlinopathies	Myonecrosis, repair defects	Membrane repair impairment by dysferlin loss and complement activation	Macrophages-driven inflammation; NF-kb signaling involved
Congenital MDs	Early onset weakness, fibrosis	NF-κB and TLR pathways activation by Laminin α-2 deficiency	Macrophage-dominated inflammation
Facioscapularhumeral MD (FSHD)	Face, shoulder weakness	DUX4 activation-related toxicity, immune activation	Muscle infiltrating T-cells and macrophages
Emery-Dreifuss MD (EDMD)	Weakness, joint contractures, cardiopathy	Nuclear defects (emerin, lamin A/C) trigger to inflammation	TNF-α, IL-6, macrophages, T-cells involved
Myotonic Dystrophy (MD)	Muscle wasting, cardiac complications	Inflammation caused by RNA toxicity from repeat expansions	Pro-inflammatory cytokines, macrophage infiltration
Oculopharyngeal MD (OPMD)	Ptosis, swallowing issues, limb weakness	Nuclear aggregates activate immune responses	Macrophages, CD8^+^ T-cells mediate fibrosis

**Table 2:** Stem cell types, sources, and roles in muscular dystrophy therapy.

Stem Cell Type	Characteristics	Source	Applications
MSCs	Immunomodulatory potential, tissue repair support, anti-fibrotic properties	Bone marrow, adipose tissue, perinatal tissues	Inflammation and fibrosis reduction in (DMD) models
AECs	Immunoprivilege, anti-inflammatory cytokine secretion (e.g., IL-10)	Epithelial layer of the amniotic membrane	Muscle regeneration enhancement and fibrosis reduction
WJ-MSCs	High stemness, low teratoma risk, potent immunomodulation	Umbilical cord (Wharton’s jelly)	Angiogenesis promotion, muscle repair, anti-inflammatory macrophage activation
BM-MNCs	Myogenic potential	Bone marrow aspirates	Use for muscle regeneration, limited efficacy in chronic inflammation stages of MD
iPSCs	Unlimited proliferation, personalized regenerative potential	Reprogrammed somatic cells (e.g., skin)	Promising for future MD treatments, limited viability and immune rejection challenges
Mesoangioblasts	Vessel-associated with myogenic differentiation ability	Large blood vessels (e.g., aorta)	Muscle fibers integration for regeneration in DMD preclinical models

## Abbreviations

AECs	Amniotic Epithelial Cells
BM MNCs	Bone Marrow Mononuclear Cells
BM-MSCs	Bone Marrow-Mesenchymal Stem Cells
CD133^+^ Cells	Hematopoietic Stem/Progenitor Cells Expressing CD133
CMDs	Congenital Muscular Dystrophies
DM	Myotonic Dystrophy
DMD	Duchenne Muscular Dystrophy
EDMD	Emery-Dreifuss Muscular Dystrophy
ESCs	Embryonic Stem Cells
FSHD	Facioscapulohumeral Muscular Dystrophy
hAMSC	human amniotic mesenchymal stem cell
HDAC inhibitors:	Histone deacetylase inhibitors
HLA	Human Leukocyte Antigen
IFNγ	Interferon Gamma
IL-10	Interleukin 10
IL-1β	Interleukin 1 Beta
IL-4	Interleukin 4
IL-6	Interleukin 6
iNOS	Inducible Nitric Oxide Synthase
LGMD R5	Limb-Girdle Muscular Dystrophy Type R5
LGMD R9	Limb-Girdle Muscular Dystrophy Type R9
LGMD	Limb-Girdle Muscular Dystrophy
LPS	lipopolysaccharide
MABs	mesongioblasts
MAC	Membrane Attack Complex
MDC1A	Merosin-Deficient Congenital Muscular Dystrophy
MDs	Muscular Dystrophies
MDSCs	Muscle-Derived Stem Cells
MSCs	Mesenchymal Stem Cells
MuSC	muscle stem cell
NF-κB	Nuclear Factor Kappa B
OPMD	Oculopharyngeal Muscular Dystrophy
OS	Oxidative Stress
PGE2	Prostaglandin E2
PL-MSCs	placenta-derived mesenchymal stem cells
ROS	Reactive oxygen species
SP Cells	Side Population Cells
TGF-β	Transforming Growth Factor β
TLR	Toll-like Receptor
TNF-α	Tumor Necrosis Factor Alpha
TSG-6	Tumor Necrosis Factor-Stimulated Gene-6
WJ-MSCs	Wharton’s Jelly-Mesenchymal Stem Cells
